# Advances in sequencing and key character analysis of mango (*Mangifera indica* L.)

**DOI:** 10.1093/hr/uhac259

**Published:** 2022-11-21

**Authors:** Miaoyu Song, Haomiao Wang, Zhiyi Fan, Hantang Huang, Huiqin Ma

**Affiliations:** College of Horticulture, China Agricultural University, Beijing 100193, China; College of Horticulture, China Agricultural University, Beijing 100193, China; College of Horticulture, China Agricultural University, Beijing 100193, China; College of Horticulture, China Agricultural University, Beijing 100193, China; College of Horticulture, China Agricultural University, Beijing 100193, China; State Key Laboratory of Agrobiotechnology, China Agricultural University, Beijing 100083, China

## Abstract

Mango (*Mangifera indica* L.) is an important fruit crop in tropical and subtropical countries associated with many agronomic and horticultural problems, such as susceptibility to pathogens, including powdery mildew and anthracnose, poor yield and quality, and short shelf life. Conventional breeding techniques exhibit significant limitations in improving mango quality due to the characteristics of long ripening, self-incompatibility, and high genetic heterozygosity. In recent years, much emphasis has been placed on identification of key genes controlling a certain trait through genomic association analysis and directly breeding new varieties through transgene or genotype selection of offspring. This paper reviews the latest research progress on the genome and transcriptome sequencing of mango fruit. The rapid development of genome sequencing and bioinformatics provides effective strategies for identifying, labeling, cloning, and manipulating many genes related to economically important traits. Preliminary verification of the functions of mango genes has been conducted, including genes related to flowering regulation, fruit development, and polyphenol biosynthesis. Importantly, modern biotechnology can refine existing mango varieties to meet the market demand with high economic benefits.

## Introduction

Mango (*Mangifera indica* L.) is a juicy drupe of Mangifera of Anacardiaceae. It is the world’s third most planted tropical fruit after banana and pineapple (http://www.fao.org/faostat/), widely grown in tropical and subtropical marginal areas [1]. The main planting areas are hillsides, river valleys, or wilderness forests at an altitude of 200–1350 meters in India, China, Thailand, Myanmar, Bangladesh, and Malaysia. The popularity of mango can be attributed to its attractive taste, fragrance and high nutritional value. The fruit consists of pulp, peel, and kernel. The pulp is rich in reducing sugars, amino acids, aromatic compounds, and functional compounds such as: pectin vitamins, anthocyanins, and polyphenols [2]. The β-carotene in mango flesh is as high as 200 mg/100 g, which is 10 and 50-fold higher than in bananas and apples. Mangiferin is the main active component in mango leaves and helps to scavenge oxidative free radicals and participate in antibacterial and immunomodulation [3, 4].

There are more than 1000 mango cultivars worldwide [5, 6]. According to its embryo type, mango fruit can be divided into monoembryonic and polyembryonic varieties. The seeds of the monoembryonic (India) variety have only one zygotic embryo, which is propagated by sexual reproduction, and only one seedling after sowing. The seedlings exhibit great variability but do not maintain the excellent characteristics of the female parent, mainly distributed in subtropics, and the peel is mostly red. In contrast, the polyembryonic (Southeast Asia) type is most common in the tropics, and the polyembryonic traits are often dominant. The polyembryonic types are produced from mother plants, and several seedlings can grow after sowing. Embryos that can develop into seedlings are mostly asexual, accounting for thesmall variability of fruiting trees, and most can preserve the traits of mother plants. The pericarp is mainly green to yellow, and Thai mango (*Mangifera siamensis* warbg. ex Craib) mostly belongs to this type [7–9]. With the development of mango variety breeding, thehybridization between monoembryonic and polyembryonic typescan yield polyembryonic offspring.

Evaluating and protecting natural mango germplasm resources is essential, and new varieties are warranted for the modern market and commercial needs. Most mango plants are heteroecious and cross-pollinated. According to literature records, the Indian Agricultural Research Association (IARI) first conducted breeding research to improve mango varieties in 1961. At present,seed selection, cross-breeding, and mutation breeding remain the main breeding ways. Due to the long juvenile phase of perennial fruit trees, the selection of mango breeding offspring by theabove methods is time-consuming and requires a lot of screening work, and the characteristics of hybrid offspring are often significantly different. Plant biotechnology provides a new approach forimproving varieties and developing stable and efficient genetic transformation methods. It has become an important meansfor genetic improvement and germplasm resource innovation of mango fruit.

Over the years, the mango hybrid offspring of different parent varieties have been used to construct genetic populations. Twenty-seven hybrids of Tommy Atkins, Haden, Palmer, Coquinho, Kent, and Van Dyke varieties have been used to estimate quality genetic parameters by modeling [10] and evaluating mango hybrids obtained through open pollination based on physical and chemical traits of the fruit [11]. Hybrid populations can help in genetic diversity analysis with significant emphasis placed on quantitative trait locus and marker-assisted selection. The offspring of JinHwang and Irwin was used to construct the first high-density genetic map for high-throughput sequencing and specific-locus amplified fragment (SLAF) library construction [12]. Interestingly, mining of RAPD primers in Indian mango hybrids has recently been conducted [13]. Besides, new hyper-variable mango SSRs (MSSRs) designed from Amrapali genome sequences have been used to discover polymorphisms between Amrapali and Sensation parental genotypes [14], and single nucleotide polymorphism (SNP) markers to genotype mango hybrid populations [15].

Early trait prediction is essential to shorten the breeding process. Molecular markers are genetic markers based on the polymorphism of biological macromolecules, especially the genetic material (nucleic acid) of organisms. They are directly expressed in the form of DNA and are not interfered with by tissue types, development periods, environmental conditions, etc. Compared with the morphological traits studied by traditional genetics and breeding, they exhibit obvious advantages, mainly reflected in the fact that the stage of plant development, gene expression and environmental changes do not affect the selection of target traits, easier and faster to overcome undesirable trait linkage and introduce distant superior genes [16]. Over the years, simple sequence repetitions (SSRs) markers [17], cleavage amplification polymorphic sequence markers [18], and reverse transposon insertion polymorphic markers [19] have been widely used in mango research. The past decade has witnessed significant progress and heralded the era of genome breeding. The key genes controlling a certain trait can be located by high-throughput sequencing and association analysis, and new varieties are bred directly by transgene or genotype selection of offspring. Accordingly, the *de novo* transcriptome data [20–22] and the genetic map data [12, 23] of mango have been successively published. In 2020, Wang *et al.* conducted high-depth whole genome sequencing of mango and documented the whole genome data at the chromosome level [22]. Modern biotechnology is an effective adjunct to traditional mango breeding. This paper will focus on the present situation and prospect of genetic improvement of mango using molecular and biotechnology. Importantly, more advanced biotechnology tools and synthetic biology will provide efficient gene editing means for improving agronomic mango traits in the future.

## Whole genome and transcriptome sequencing

### Fine-scale genomic map of mango

Before second generation sequencing technology was first used to sequence mango in 2014, mango genome sequence resources were very scarce, with only 684 highly redundant sequence entries in the GenBank. Azim *et al.* first sequenced, assembled, and annotated the mango chloroplast genome. The chloroplast genome of mango was 151, 173 bp in size, comprising a pair of reverse repeats of 27, 093 bp, separated by large and small single copies. A total of 91 out of 139 genes in the mango chloroplast genome were protein-coding genes. Sequence analysis showed that the chloroplast genome of citrus was closest to that of mango [24]. To better understand the basic molecular biology of mango fruit, large-scale discovery and characterization studies of functional genes by genome sequencing or transcriptome have been carried out worldwide. In 2018, Qamar-ul-Islam *et al.* reported four mango varieties – Cv. Langra, Cv. Zill, Cv. Shelly, and Cv. Kent – and, moreover, conducted a comparative analysis of the transcriptome [25]. This paper reports the world’s first online genome resource focusing on mango. It contains predicted gene information of the whole genome, unigenes annotated by homologous genes of other species, and Gene Ontology (GO) terms, providing a mango genome resource and allowing users to analyse the genome database of four mango varieties for genetic improvement and management of mango genome [25].

Although mangoes are highly heterozygous, the current evidence suggests that the mango germplasm is diploid (2n = 2x = 40 chromosomes) [7, 26]. In this respect, in 1991, Arumuganathan *et al.* analysed the nucleus DNA content of mango in their study using flow cytometry with propidium iodide staining of isolated nuclei, it was proposed that the genome size of mango is about 4.39 × 10^8^ bp [27]. Besides, the number of chromosomes in mango somatic cells was determined by the Carbol fuchsin method to be 2n = 40, and no individuals with other ploidy were found [28, 29]. The polyembryonic mango ‘Gomera-1’ has been confirmed to be diploid by flow cytometry and chromosome count analysis [30]. Yonemori *et al.* conducted the first study using fluorescence in situ hybridization (FISH) technique with 5S and 45S ribosomal DNA (rDNA) as probes on the mid chromosomes of mango somatic cells and discriminated 8 out of 40 chromosomes [31]. This information provides a basis for understanding the number of chromosomes mounted during sequencing.

**Figure 1:**
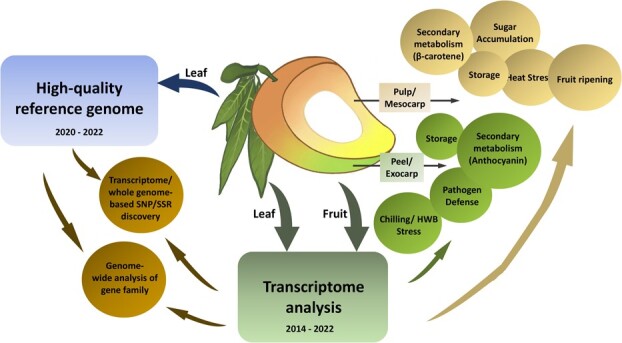
Genomic approaches in Mango.

In 2020, the first mango genome was published, providing a fine-scale mango genome map [22] with a size of 393 Mb by deep sequencing and assembly of data on the traditional mango variety Alphonso. From 2020 to 2022, genome and transcriptome analyses have been used to analyse variations in gene sequence and gene expression and specifically applied to fruit development-related research in mango (Fig. 1, Table 1). At the same time, Li *et al.* obtained a 371.6 Mb genome from Hong Xiang Ya mango by SMRT sequencing, which contained 34 529 predictive protein-coding genes, providing the genetic basis for understanding special phytochemical compounds related to fruit quality [32]. Ma *et al.* sequenced Irwin varieties and obtained a high-quality genome sequence of 396 Mb. After transcriptome analysis, they found that the transcriptional regulation of the MiPSY1 gene was related to β-carotene biosynthesis during mango fruit ripening, which provided a genome platform for studying the molecular basis of mango flesh color [33]. The Mango Genome Consortium (https://mangobase.org/easy_gd b/index.php) sequenced, recombined, analysed, and annotated the genome of the monoembryonic mango variety Tommy Atkins and used the hybrid between Tommy Atkins and Kensington Pride to generate phased haplotype chromosomes and a high-resolution phased single nucleotide polymorphism map, beneficial to identify quantitative trait loci (QTL), gene and haplotype related to fruit weight [34]. In 2022, Cortaga *et al.* determined the whole genome sequences of three Philippine mango species (Carabao, Huani, and Paho) for identifying genome-wide specific markers for these Philippine native mango varieties [35]. This genomic information can guide future research to better understand the growth, survival status, and gene regulation mechanisms of mango.

**Table 1:** Mango genome resources

**Cultivars**	**Tissues**	**Direction**	**Approach/Methods**	**Estimated genome size**	**Predicted genes**	**Results**	**Reference**	**Accession number**
Langra	Leaves	Chloroplast DNA	Illumina HiSeq2000/Sanger	151,173 bp	139	Circular map of the mango chloroplast genome with 30 notches.	(Azim *et al.* 2014) [24]	NCBI project ID: FJ212316
Alphonso	Leaves	High-quality reference genome	PacBio/Illumina Hiseq3000	392.9 Mb	41 251	Complete genome assembly of mango chromosome.	(Wang *et al.* 2020b) [22]	NCBI project ID: PRJNA487154
Hong Xiang Ya	Leaves	High-quality reference genome	PacBio/Illumina Hiseq4000	371.6 Mb	34 529	High-quality genome with a 98.77% chromosome assembly rate	(Li W 2020) [32, 33]	NCBI project ID: PRJCA002248
Irwin	Leaves	High-quality reference genome	PacBio/Illumina HiSeq Xten	396 Mb	36 756	To explore the molecular basis of pulp color regulation with the transcriptome.	(Ma *et al.* 2021) [33]	GSA in the national genomics data center: CRA004336
Tommy Atkins	Leaves	High-quality reference genome	HiSeqX/Hiseq2500/HiSeq4000	377 Mb	26 616	To locate QTL regions related to commercial fruit size.	(Bally *et al.* 2021) [34]	NCBI project ID: PRJNA450143

### Transcriptome sequencing analysis of mango

As shown in Fig. 1 and Table 2, current mango transcriptome research has mainly focused on the process of fruit development. Fruit ripening is a complex process during which the development of flavor and color, the change and softening of cell wall components, the degradation of starch, and the development of aroma occur and determine the unique fruit characteristics. In 2014, Azim *et al.* used the short-read assembly program Trinity for *de novo* transcriptome assembly of Langra mango, which was the first report on the transcriptome of Rhizaceae plant members. More than 13 500 unigenes were assigned to 293 KEGG pathways. In addition to the main pathways related to plant biology, KEGG pathway analysis revealed significant enrichment in a series of biochemical pathways involving (i) biosynthesis of bioactive flavonoids, flavonoids and flavonols; (i) biosynthesis of terpenoids and lignin; and (iii) plant hormone signal transduction, providing novel insights into exploring key regulatory genes in mango growth and development by transcriptome technology [24].

**Table 2:** Mango transcriptome resources

**Cultivars**	**Tissues**	**Direction**	**Approach/Methods**	**Multi-omics**	**Predicted genes**	**Results**	**Reference**	**Accession number**
Langra	Leaves	Secondary metabolism	Illumina HiSeq2000/ de novo		30 509 unigenes	Gene annotations provide information on the production of flavonoids, carotenoids and terpenoids.	(Azim *et al.* 2014) [24]	NCBI project ID: SUB363843
Zill	Fruit	Development and ripening	Illumina HiSeq2000/ *de novo*	Proteomic	54 207 transcripts	Revealed candidate genes/proteins involved in fruit development and ripening.	(Wu *et al.* 2014) [36]	NCBI project ID: SRP035450
Shelly	Peel	Stress: hot water brushing	Illumina HiSeq2000/ *de novo*		57 544 unigenes	Mango fruit quality improvement after heat treatment is a synergistic effect of multiple stress response mechanisms.	(Luria *et al.* 2014) [37]	NCBI project ID: SRX375390
Kent	Mesocarp	Fruit ripening	Genome Analyzer GAIIx II/*de novo*		52 948 unigenes	The data reflect the changes at the transcriptional level during mango ripening.	(Dautt-Castro *et al.* 2015) [38]	NCBI project ID: SRP045880
Zill	Peel	Defense response	Illumina HiSeq2000/ *de novo*		131 750 unigenes	Identification of ERFs, NBS-LRRs, NPRs and PRs genes associated with defense responses with anthracnose.	(Hong *et al.* 2016b) [39]	No information
Keitt	Peel	Stress: chilling	Illumina HiSeq2000/ *de novo*		57 576 unigenes	To elucidate the molecular basis of response to chilling injury.	(Sivankalyani *et al.* 2016) [40]	NCBI project ID: SRP066658
Alphonso	Flower and fruits	Development and ripening	Illumina HiSeq2000/ *de novo*		434 366 transcripts	Flavor, color, ripening time, ripening pattern and shelf life affect the transcriptional characteristics.	(Deshpande *et al.* 2017) [41]	NCBI project ID: PRJNA391381
Keitt	Peel	Development and storage	Illumina HiSeq2500/ *de novo*		107 744 unigenes	Identification of mango fruit cuticle biosynthesis gene.	(Tafolla-Arellano *et al.* 2017) [21]	NCBI project ID: SRP043494
Ataulfo	Peel	Stress: hot water brushing	Illumina Genome Analyzer GAIIx II/ *de novo*		54 379 transcripts	Deepening the understanding of the genes and pathways controlling mango fruit softening triggered by HWT.	(Dautt-Castro *et al.* 2018) [42]	NCBI project ID: PRJNA286253
Amrapali	Peel	Secondary metabolism	Illumina NextSeq 500/MiSeq/*de novo*		43 037 unigenes	Fifteen transcripts involved in anthocyanin biosynthesis.	(Bajpai *et al.* 2018) [43]	NCBI project ID: SRP070908
Mango cv. 1243	Tissue pool	Comprehensive RNA-Seq datasets	Illumina NextSeq 500/ *de novo*		82 198 unigenes	Provide transcriptome resources for mango fruit developmental gene expression.	(Chabikwa *et al.* 2020) [44]	NCBI project ID: PRJNA533518
Chaunsa White	Pulp	Stress: heat	5500 SOLiD/*de novo*	Metabolomic	107 744 unigenes	Heat stress induced the synthesis of ROS, activated the antioxidant defense mechanism and accelerated fruit ripening speed.	(Khanum *et al.* 2020) [45]	http://bioinfo.bti.cornell.edu/cgi-bin/mango/index.cgi
Tainong No. 1 Renong No. 1	Pulp	Sugar accumulation	Illumina HiSeq2500/ *de novo*	Metabolomic	256 774 unigenes	The synergistic effect of MYB and NAC with key genes of sucrose transport/metabolism/synthesis is the main reason for the high sugar content.	(Li W 2020) [32]	NCBI project ID: PRJNA629065
Neelam, Dashehari	Leaves	Alternate bearing	5500 SOLiD/*de novo*		42 397 unigenes	Potential candidate genes related to hormone and carbohydrate pathways.	(Sharma *et al.* 2020) [46]	NCBI project ID: SRR1297075, SRR1298995
Tainong	Pulp	Development and storage	Illumina HiSeq2500/ *de novo*	volatile profile, metabolomics	53 361 unigenes	Molecular determinants of aroma component synthesis during mango fruit development and storage.	(Xin *et al.* 2021) [47]	NCBI project ID: PRJNA697524

Fruit-specific secondary metabolites and aroma volatiles are important markers to distinguish between immature and mature stages, such as carotenoids, anthocyanins, and aroma in mango fruits. Transcriptome analysis of Alphonso mango fruit was used to analyse the unique transcription profile characteristics affecting fruit flavor, color, ripening time, ripening pattern from peel to the core and long shelf life [48]. The transcriptome analysis of Amrapali mango was used to clarify the transcription trend of key genes related to peel color in the anthocyanin biosynthesis pathway. Among the 108 transcription sequences of the phenylpropanoid flavonoids pathway, 15 contigs were identified as anthocyanin biosynthesis genes [43]. To explore the molecular basis of mango flavor formation, the molecular determinants of carotenoid and aroma composition in mango [20, 47] were explored using volatile spectrum, metabonomics, and transcriptomics. The MYB, bHLH, and NAC transcription levels were highly correlated with pulp pigment content, which may be related to carotenoid accumulation. This finding highlights the main differences in metabolic pathways during fruit ripening, which may lead to a change in mango fruit flavor, and reveals several related genes for future studies. The discovery of transposon-mediated ncRNA in crops has facilitated analysis of metabolic regulation in mango fruit, with around 100 miRNA and more than 7000 temperature-responsive lncRNA. Interestingly, some lncRNA-targeted miRNA could reduce the stability of lncRNA, and the target genes of these ncRNA were characterized. The newly identified mango ncRNAs may play potential roles in biological and metabolic pathways such as growth and development, pathogen defense mechanism, and stress response process [49].

Sweetness is an important trait that determines fruit quality. It is well-established that during mango flesh ripening, starch is hydrolyzed into sucrose, fructose, and glucose with different concentrations catalyzed by invertase and β-glucosidase, which account for the unique sweet taste of mango varieties. Transcriptome analysis of differences in sugar accumulation between the high-sweet mango Tainong-1 and low-sweet Mango Renong-1 found that the key genes exerted a synergistic effect in sucrose transport, metabolism, and biosynthesis through regulating transcription factors such as MYB and NAC was the main reason for high sugar content, but no specific regulatory gene was identified [32].

Abiotic and biotic stresses are important factors affecting fruit ripening. Environmental stress conditions such as drought, salinity, high temperature, and flood can significantly interfere with the development and yield of tropical fruit trees and affect the fruit quality. Ripening can change the hardness of fruits, making them vulnerable to pests and pathogens in the final stages of ripening or during storage [50]. Gene expression analysis was used to clarify the biological mechanism of hot water brushing (HWB) activation in mango regulating fruit quality [42] and resistance to postharvest diseases [37]. The results showed that a high temperature could induce internal tissue decomposition of mango fruit and synthesis of reactive oxygen species (ROS) at 44°C and increase the expression of abiotic and senescence-related genes in mango fruit in response to heat stress [37, 45]. In a study on disease resistance and defense genes, Hong *et al.* obtained the first reference transcriptome data of *Colletotrichum gloeosporioides* in postharvest mango by high-throughput next-generation sequencing technology [39]. In the same year, Sela *et al.* discovered a new *M. indica* latent virus (MILV) virus sequence for whole transcriptome sequencing of mango fruit. Although no virus-related symptoms were detected, the differential gene analysis of mango peel transcriptome showed significant stress in mango peel, and the gene expression related to plant immune response to pathogen and virus infection increased [51].

## Analysis of important characteristics of mango using genome and transcriptome data

### Genomic assisted breeding

Sequencing technology ranging from genetic diversity analysis and DNA fingerprinting with molecular markers to high-throughput sequencing based on SNP, target region amplification polymorphism (TRAP), and SSR markers has led to the development of the fruit tree genome. The development of genetic markers based on mango genome or transcriptome is shown in Table 3. In 2015, Sherman *et al.* used Illumina sequencing technology for the first time to sequence Keitt and Tommy Atkins cultivars. A total of 332 016 single nucleotide polymorphisms (SNPs) and 1903 SSRs were found, and polymorphism in Israeli mango was assessed, indicating that transcriptome data analysis can significantly broaden the genetic variation data of mango fruit [52]. Using next-generation sequencing technology, RNA sequences of mango parent varieties Neelam, Dashehari, and their hybrid varieties Amrapali were analysed. The *de novo* sequence assembly generated 27 528, 20 771, and 35 182 transcripts, respectively, and further assembled into 70 057 non-redundant unigenes for SSR and SNP identification and annotation [16]. The main advantage of developing molecular markers based on the transcriptome sequences is to increase the possibility of finding associations between functional genes and phenotypes and analysis of key traits, including fruit size, fruit flavor and storability of hybrid offspring [16]. The first high-density genetic map of Mango was constructed by high-throughput sequencing of 173 F1 lines hybridized by JinHwang and Irwin: 6594 SLAFs were organized into a linkage map consisting of 20 linkage groups and were conducive to future genome assembly [12]. The discovery of RAD-based markers improves the development of network genome resources for plant genetic improvement and germplasm management and identifies SNP in the whole genome [53, 54]. A total of 28.6 Gb data of 84 mango varieties were generated by Iquebal *et al.* using the ddRAD-Seq method on Illumina HiSeq 2000 platform, and a total of 1.25 million SNP data were found. In addition, Warschefsky *et al.* analysed 158 mango samples, of which 106 were from known mango varieties and 52 were from related species or unknown varieties for developing high-density linkage maps, QTL discovery, variety differentiation, traceability, genome collation and SNP chip development, which provided a reference for the GWAS genome selection program [55, 56]. Kuhn *et al.* screened 500 000 SNP markers from RNA sequencing and transcriptome comparison [52, 57], making genetic maps to identify genomic markers and regions related to important horticultural traits of mango (such as embryo type, branching habit, flowering, peel/pulp color, and beak shape) [23], and further improving breeding efficiency. Genome-assisted breeding provides the necessary resources for developing high-density and cost-effective genotyping research, which is of great help to mango breeding and genome-wide association of yield and quality traits.

**Table 3:** Development of genetic markers based on genome/transcriptome

**Cultivars Tissues**		**Direction**	**Approach/** **Methods**	**Reference genomes**	**Variation**	**Results**	**Reference**	**Accession number**
Kensington Pride, Irwin, Nam Doc Mai		Variability in genes		EST database	SNP	To identify flavonoid synthesis genes and facilitate the characterization of SNP among cultivars.	(Hoang *et al.* 2015) [57]	http://mango.qfab.org
Tommy Atkins, Keitt	Tissue pool	Align resequencing	454-GS FLX Titanium/*denovo*	Keitt reference-transcriptome contigs	SNP, SSR	A large pool of genetic variation has been established in mango.	(Sherman *et al.* 2015) [52]	NCBI project ID: PRJNA254771
Neelam, Dashehari, Amrapali	Leaves	Hybrid varieties heterozygosity	Illumina HiSeq2000/*de novo*	Unigene set	SNP, SSR	The heterozygosity of SNP in hybrid Amrapali was significantly enhanced.	(Mahato *et al.* 2016) [16]	NCBI project ID: PRJNA193591, PRJNA193588, PRJNA279829
Jin-Hwang, Irwin	Leaves	The first high-density genetic map	Illumina HiSeq 2500	SRA database	SNP	Used to identify germplasm or hybrids and to analyse genetic diversity among cultivars.	(Luo *et al.* 2016) [12]	NCBI project ID: SRX1741570
84 varieties	Leaves	Web-based genomic SNPs resources	Illumina HiSeq2000/ddRAD-Seq/*de novo*		SNP	MiSNPDb resources	(Iquebal *et al.* 2017) [55]	http://webtom.cabgrid.res.in/mangosnps/
24 mango cultivars	Leaves	SNP-based genetic markers	Illumina HiSeq2000/*de novo*	Tommy Atkins transcriptome sequence	SNP	The SHRS SNP marker was used for genotyping seven different populations (775 individuals) of the extant mango mapping population.	(Kuhn *et al.* 2017) [23]	No information
106 cultivars	Leaves	Domestication history	Illumina HiSeq2500/*de novo*	Three ddRADseq libraries	SNP	Two cultivated mango gene banks representing Indian and Southeast Asian germplasm were identified.	(Warschefsky & von Wettberg 2019) [56]	NCBI project ID: PRJNA517351
Carabao, Huani, Paho	Leaves	High-quality reference genome	Illumina HiSeq 2500	Alphonso, Tommy Atkins	SNP, InDel	Revealed genome-wide variation	(Cortaga *et al.* 2022) [35]	NCBI project ID: PRJNA740276

### Functional verification of genes related to flowering regulation in mango

Using omics data, researchers have focussed on studying functions at the molecular level, identifying genes through annotation, studying expression regulation mechanisms and functions in metabolic pathways of organisms, analysing the relationship between genes and products, and predicting and discovering protein functions. Current transcriptomics research of mango fruit has mainly focused on the fruit, while the functional verification of genes at physiological and biological levels has focused on flowering regulation (Table 4), fruit development, and metabolite synthesis (Table 5).

**Table 4:** Functional validation of genes related to flowering regulation in mango based on transcriptome

**Cultivars**	**Gene**	**Genetic transformation**	**Function**	**Proven** **target genes**	**Reference**	**Reference** **RNA-seq**	**Gene ID**
Dashehari, Amrapali	*MiFT*		Negative relation with GA	MiAP1	(Das *et al.* 2019; Nakagawa *et al.* 2012) [58,59]		KX093179
Carabao	*MiSOC1*		Induce flower		(Wei *et al.* 2016) [60]		KP404094
SiJiMi, TaiNong No. 1	*MiCO*	*Arabidopsis*	Delay flowering under long-/ short-day conditions		(Liu *et al.* 2020) [61]	Unpublished data	HQ585995, JQ700253
SiJiMi	*MiFT1*	*Arabidopsis*	Promoting flowering		(Fan *et al.* 2020) [62]	Unpublished data	MT419778
	*MiFT2*						MT419779
	*MiFT2*						JQ700254
SiJiMi	*MiCOL1A*		Flowering regulation and stress response		(Guo *et al.* 2022a) [63]	Unpublished data	Unpublished data
Amrapali,	*MiGA(20)OX3*		Regulate flower development and malformations		(Yadav *et al.* 2020) [64]	SAMN05727981,SAMN05727982, SAMN05727983, SAMN05727984,	CDS_24125_Unigene_25942
	*MiAGL24*						CDS_26385_Unigene_29342
	*MiLDL2*						CDS_18357_Unigene_21030:7.0)
SiJiMi, TaiNong No. 1	*MiAP1-1*	*Arabidopsis* *Tobacco*	In floral transition and organ development.		(Yu *et al.* 2020) [65]		No. GQ152892
	*MiAP1-2*						No. GQ152893
Ratna	*MiGI2*		Temperature-dependent floral induction	MiFKF1,MiCDF1, MiCO	(Patil *et al.* 2021) [66]		MZ357241
SiJiMi	*MiTFL1-1*	*Arabidopsis*	In the flowering process	MibHLH13/162,14-3-3,MiFD	(Wang *et al.* 2021) [67]	Unpublished data	AGA19021.1
	*MiTFL1-2*						AGA19021.2
	*MiTFL1-3*			MibHLH13			AGA19021.3
	*MiTFL1-4*			MibHLH162			AGA19021.4
SiJiMi	*MiSVP1*	*Arabidopsis*	In the flowering process	MiSEP1-1, MiSOC1D, MiAP1-2	(Mo *et al.* 2021b) [68]	Unpublished data	MZ542518
	*MiSVP2*						MZ542519
SiJiMi	*MiCOL1B*	*Arabidopsis*	Flowering regulation and stress response		(Guo *et al.* 2022a) [63]	Unpublished data	Unpublished data
SiJiMi	*MiCOL16A*	*Arabidopsis*	Flowering regulation and abiotic stress response	AtFT, AtSOC1	(Liu *et al.* 2022) [69]	Unpublished data	Unpublished data
	*MiCOL16B*						
SiJiMi,TaiNong No. 1	*MiLFY*	*Arabidopsis*	Flowering regulation	MiZFP4, MiSOC1D	(Wang *et al.* 2022) [70]	Unpublished data	HQ585988
Carabao	*MiSEP1*		Flowering regulation		(Wei 2017) [71]		KP702299
Dashehari	*MiErpA1*		Ripening		(Sane *et al.* 2005) [72]		No. AY600964
	*MiCel1*		Ripening and softening		(Chourasia *et al.* 2008) [73]		No. EF608067.
Kent	*MiERS1*		Regulating fruitlet abscission, prolonging storage life		(Ish-Shalom *et al.* 2011) [74]		JF323582
	*MiETR1*						AAF61919.1
Zill	*MiExpA1*		Ripening and softening		(Zheng *et al.* 2012) [75]		No. AY600964
Kent	*MiIDA1*		Fruitlet abscission		(Rai *et al.* 2021) [76]		QGF19396.1
	*MiIDA2*						QGF19397.1
Alphonso, Pairi, Kent	*Mi9LOX*		Lactone biosynthesis		(Deshpande *et al.* 2017) [41]		KX090178
	*MiEH2*						KX090179
Irwin	*MiCHS*		Anthocyanin biosynthesis		(Kanzaki *et al.* 2019) [77]		
	*MiANS*						
	*MiUFGT1,3*						No.: LC47860/1/2
Guiqi, Jinghuang, Guifei	*MiPAL*		Anthocyanin biosynthesis		(Zhao *et al.* 2022) [78]		GU266281.1
Hongmang No. 6, Sensation, Geifei, Jinhuang, Qingmang	*MiWRKY1,3,5,81,84,*		Anthocyanin biosynthesis		(Shi *et al.* 2022) [79]	PRJNA48715	mango033727.t1, mango015757.t1, mango000102.t1, mango027343.t1,mango029640.t1
Tainong 1, Hongyu, Kaituk, Nam Dok Mai No. 4, Nam Dok Mai Sithong.	*MiPSY*		Carotenoid accumulation	MiZDS, MiBCH MiZEP	(Ma *et al.* 2018; Yungyuen *et al.* 2021) [80,81]		XM_044650327.1
Chokanan Golden, Phoenix, Water lily	*MiRab3*		Participates in plant physiological processes, including fruit ripening.		(Lawson et al. 2020) [82]		Z71276.1 KF768563
	*MiRab5*						
Kent	*MiLAX2*		Auxin-related gene		(Denisov *et al.* 2017) [83]	SAMN02905156	
	*MiPIN1*					SAMN02947194	
Hôi	*MiERS1a*	*Arabidopsis*	Positively responsive to ethylene		(Winterhagen *et al.* 2019) [84]		KU886218
	*MiERS1b*						KU886217
Zill	*MiACO*		SA and NO signal induction		(Hong *et al.* 2014) [85]		AY700081.1
	*MiACS*						AY700086.1
	*MiERS1*						KU886218.2
Siji	*MieIF1A-a*	*Arabidopsis*	Enhance the salt tolerance		(Li *et al.* 2019b) [86]		KP271044
	*MieIF5*						MK002432
	*MieIF3sB*						MK002421
SiJiMi	*MiTTG1*	*Arabidopsis*		MiMYB0, MiTT8 and MibHLH1	(Tan *et al.* 2021) [87]	PRJNA487154	Mi01g20920
SiJiMi	*MiSPLs*	*Arabidopsis*	Promot tolerance to drought, ABA and GA3		(Zhu *et al.* 2022) [88]	unpublished data	LOC123215790
SiJiMi	*Mi14-3-3*		Plays an important role in the stress of mango		(Xia *et al.* 2022) [89]	unpublished data	OK491862-73, OK203791-92 OK491860-61
Jinhuang	*MiNBS-LRR*		Recognize pathogenic virulence factors		(Lei *et al.* 2014) [90]		HM446507-22
Chok, Tainong No.1	*MiACT1*		Internal standard		(Luo *et al.* 2013) [91]		JF737036

**Table 5:** Functional validation of genes related to mango fruit quality

**Cultivars**	**Gene**	**Genetic transformation**	**Function**	**Proven target genes**	**Reference**	**Reference** **RNA-seq**	**Gene ID**
Dashehari	*MiErpA1*		Ripening		(Sane *et al.* 2005) [72]		No. AY600964
	*MiCel1*		Ripening and softening		(Chourasia *et al.* 2008) [73]		No. EF608067.
Kent	*MiERS1*		Regulating fruitlet abscission, prolonging storage life		(Ish-Shalom *et al.* 2011) [74]		JF323582
	*MiETR1*						AAF61919.1
Zill	*MiExpA1*		Ripening and softening		(Zheng *et al.* 2012) [75]		No. AY600964
Kent	*MiIDA1*		Fruitlet abscission		(Rai *et al.* 2021) [76]		QGF19396.1
	*MiIDA2*						QGF19397.1
Alphonso, Pairi, Kent	*Mi9LOX*		Lactone biosynthesis		(Deshpande *et al.* 2017) [41]		KX090178
	*MiEH2*						KX090179
Irwin	*MiCHS*		Anthocyanin biosynthesis		(Kanzaki *et al.* 2019) [77]		
	*MiANS*						
	*MiUFGT1,3*						No.: LC47860/1/2
Guiqi, Jinghuang, Guifei	*MiPAL*		Anthocyanin biosynthesis		(Zhao *et al.* 2022) [78]		GU266281.1
Hongmang No. 6, Sensation, Geifei, Jinhuang, Qingmang	*MiWRKY1,3,5,81,84,*		Anthocyanin biosynthesis		(Shi *et al.* 2022) [79]	PRJNA48715	mango033727.t1, mango015757.t1, mango000102.t1, mango027343.t1,mango029640.t1
Tainong 1, Hongyu, Kaituk, Nam Dok Mai No. 4, Nam Dok Mai Sithong.	*MiPSY*		Carotenoid accumulation	MiZDS, MiBCH MiZEP	(Ma *et al.* 2018; Yungyuen *et al.* 2021) [80,81]		XM_044650327.1
Chokanan Golden, Phoenix, Water lily	*MiRab3*		Participates in plant physiological processes, including fruit ripening.		(Lawson et al. 2020) [82]		Z71276.1 KF768563
	*MiRab5*						
Kent	*MiLAX2*		Auxin-related gene		(Denisov *et al.* 2017) [83]	SAMN02905156	
	*MiPIN1*					SAMN02947194	
Hôi	*MiERS1a*	*Arabidopsis*	Positively responsive to ethylene		(Winterhagen *et al.* 2019) [84]		KU886218
	*MiERS1b*						KU886217
Zill	*MiACO*		SA and NO signal induction		(Hong *et al.* 2014) [85]		AY700081.1
	*MiACS*						AY700086.1
	*MiERS1*						KU886218.2
Siji	*MieIF1A-a*	*Arabidopsis*	Enhance the salt tolerance		(Li *et al.* 2019b) [86]		KP271044
	*MieIF5*						MK002432
	*MieIF3sB*						MK002421
SiJiMi	*MiTTG1*	*Arabidopsis*		MiMYB0, MiTT8 and MibHLH1	(Tan *et al.* 2021) [87]	PRJNA487154	Mi01g20920
SiJiMi	*MiSPLs*	*Arabidopsis*	Promot tolerance to drought, ABA and GA3		(Zhu *et al.* 2022) [88]	unpublished data	LOC123215790
SiJiMi	*Mi14-3-3*		Plays an important role in the stress of mango		(Xia *et al.* 2022) [89]	unpublished data	OK491862-73, OK203791-92 OK491860-61
Jinhuang	*MiNBS-LRR*		Recognize pathogenic virulence factors		(Lei *et al.* 2014) [90]		HM446507-22
Chok, Tainong No.1	*MiACT1*		Internal standard		(Luo *et al.* 2013) [91]		JF737036

**Figure 2:**
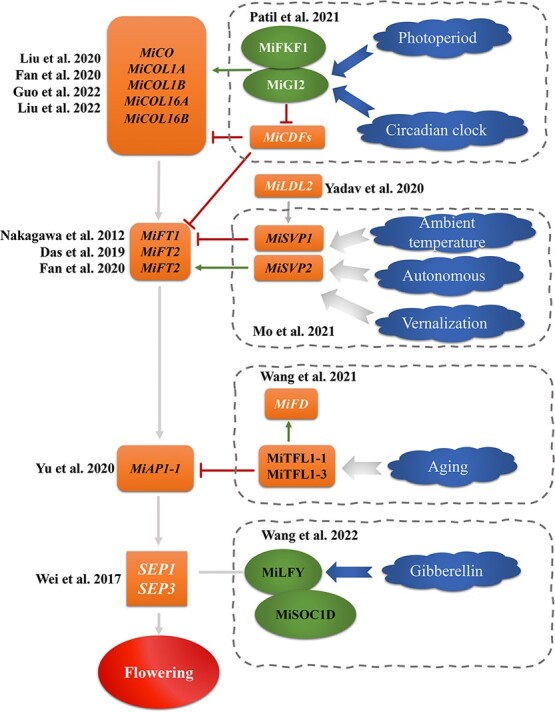
Flowering regulatory genes in mango. AP1, APETALA 1; CDF1, Cycling DOF Factor 1; CO, CONSTANS; COL1A/B, CONSTANS-like 1A/B; COL16A/B, CONSTANS-like 16A/B; FD, FLOWERING LOCUS D; FKF1, Flavin-Binding Kelch Repeat F box Protein; FT 1/2/3, FLOWERING LOCUS T 1/2/3; GI, Gigantea 2; LDL2, Lysine Specific Demethylase Like 1; LFY, LEAFY; SEP 1/3, SEPALAATA 1/3; SOC1, SUPPRESSOR OF OVER-EXPRESSION OF CONSTANS1; SVP 1/2, Short Vegetative Phase 1/2; TFL1, TERMINAL FLOWER 1. Genes in black color font have previously been validated by transgenic Arabidopsis or tobacco.

Flowering and fruit parameters play a critical role in growth and development events, and many genes and proteins related to flowering regulation have been isolated and identified in mango (Fig. 2). The GIGANTE (GI)-(FKF1)-(CDF1)-(CONSTANS) CO module has been associated with the regulation of flowering in the photoperiod and circadian pathways [92]. The expression of *MiGI* in mango is controlled by photoperiod and biological clock and forms a complex with the MiFKF1 protein to induce MiCO expression. The MiGI-MiFKF1 complex degrades MiCDFs, which inhibits the transcription of *MiCO* and *MiFT* genes [66]. Thirty-six CO homologous genes in mango were found by transcriptome data analysis [93]. Overexpression of *MiCO*, *MiCOL1A*, *MiCOL1B*, *MiCOL16A*, and *MiCOL16B* significantly delayed the flowering time of transgenic *Arabidopsis thaliana* and enhanced the drought tolerance of transgenic *A. thaliana*, which may be due to inhibition of *AtFT* and *AtSOC1* expression [61,63,69].

SVP gene is involved in mediating the environmental temperature, autonomic regulation and the vernalization pathway to regulate flowering [94]. Moreover, mango MiSVP1 overexpression in *A. thaliana* can delay flowering time by promoting *AtFLC* expression and inhibiting *AtFT* and *AtSOC1* levels, while *MiSVP2* overexpression can promote the expression of *AtFT* and *AtSOC1*, inhibit *AtFLC* expression and accelerate flowering [68]. Three *MiFTs* homologous genes in mango were only increased in leaves under optimal flower induction conditions, and treatment with 250-ppm gibberellin 3 (GA3) completely inhibited flowering and *MiFT* expression under heavy crop load and no crop load conditions [58]. Tissue-specific expression patterns showed that *MiFT1* expression increased sharply in leaves and was significantly higher than the other two *MiFTs* during flower bud development. Overexpression of three *MiFTs* in *A. thaliana* showed that MiFT1 yielded the most potent effect on promoting flowering [62].

MiTFL1 is involved in the regulation of flowering mediated by the aging pathway, and overexpression of *MiTFL1–1* and *MiTFL1–3* in *A. thaliana* can affect the development of flower organs [95]. There are four APETALA1 (AP1) homologous genes in mango, namely *MiAP1–1*, *MiAP1–2*, *MiAP1–3*, and *MiAP1–4*. *MiAP1–1* and *MiAP1–2* are highly expressed in the flowers, and overexpression in Arabidopsis significantly promotes flowering [65]. *MiAP1–2* has been associated with an early flowering phenotype in transgenic tobacco [65].

It has been reported that *MiLFY* expression could be downregulated by exogenous gibberellin (GA3) and upregulated by paclobutrazol (PPP333). Bimolecular fluorescence complementation (BiFC) experiment showed that MiLFY protein could interact with zinc finger protein 4 (ZFP4) and CONSTANS overexpression inhibitor 1 (*MiSOC1*) to promote the early flowering of *A. thaliana* [70]. Moreover, *MiSOC1* has been isolated and identified from mango. Low ethephon concentration could upregulate *MiSOC1* expression, but a high concentration inhibited *MiSOC1* expression. Overexpression of *MiSOC1* promoted the flowering of *A. thaliana* [96]. SEPALLATA (SEP) gene has been reported to be highly expressed in mango inflorescence [97]. Analysis of the distribution, phylogenetic relationship, subfamily division, gene amplification and evolution mechanism of gene families in the plant genome enables us to speculate on future gene evolution and function. Twenty-six SQUAMOSA promoter binding protein-like (SPL) family members were identified and analyzed in the ‘SiJiMi’ mango genome (unpublished data). Among them, 15 *MiSPLs* genes were highly upregulated during the early flowering stage. Overexpression of *MiSPL13* promoted the early flowering of transgenic *A. thaliana* and the expression of *AtAP1*, *AtSOC1*, and *AtFUL*, which significantly improved the tolerance to drought, abscisic acid (ABA), and GA3 and was sensitive to Pro-Ca treatment [98].

### Functional verification of mango fruit quality-related genes

Mango fruit ripening often starts at an early stage, and *MiErpA1*, *MiCel1*, *MiERS1*, *MiETR1*, and *MiExpA1* play a role in fruit ripening and softening [72–75,99]. A study found that *MiErpA1* expression was triggered within 90 min of ethylene treatment, and maturation-related transcription accumulation peaked on the third day after ethylene treatment. Importantly, 1-MCP treatment inhibited ripening/softening and *MiExpA1* transcript and protein accumulation [72]. *MiExpA1* expression may be ethylene-dependent, and its expression increases during maturation. During maturation, the accumulation of *MiCel1* transcripts gradually increases, related to increased EGase activity and decreased cellulose/hemicellulose content. The fruit ripening of control (ethylene treatment) and 1-MCP treatment was delayed by about 3 days, associated with a delayed increase in *MiCel1* expression and EGase activity [73]. Oxalic acid significantly inhibited the decrease of pulp hardness and delayed MiExpA1 expression in peel and pulp. Oxalic acid alleviated cell wall disintegration during mango fruit storage, thus delaying the softening and ripening process of mango fruit [75]. Overwhelming evidence substantiates that *MiETR1* and *MiERS1* mRNA levels are upregulated with the prolongation of storage time, peaking on day 6. 1-MCP treatment significantly decreased *MiETR1* expression on days 4, 6, and 10 and inhibited *MiETR1* expression on days 2, 4, 6, and 10. These results indicate that *MiETR1* and *MiERS1* play an important role in ethylene signal transduction. The 1-MCP treatment effectively inhibited ethylene biosynthesis and ethylene-induced maturation and senescence [99].

The ripening and softening process of fruits involves the production and transport of cell wall polymers and enzymes. It has been established that Rab guanosine triphosphatases (GTPases) are the main regulators directing traffic in the endomembrane systems. Twenty-three genes encoding RabA protein were identified using the existing mango transcriptome [21], and the relationship between pulp hardness and RabA gene expression of different mango varieties was studied, which substantiated the importance of pulp softening and transportation [82]. WRKY plays an important role in the plant defense regulatory network, development process and physiological processes such as various biotic and abiotic stress responses. Interestingly, 38 *MiWRKYs* genes correlated with mango malformation traits [100]. Compared with transcriptome analysis, searching the whole gene family through the genome can yield more comprehensive information. Polygalacturonase is a cell wall degrading enzyme that degrades pectin and participates in the softening of fleshy fruits during ripening. A total of 17 *PGs* cDNA were detected in the Kent mango peel transcriptome, while a total of 48 PGs genes were found in the mango genome, among which *MiPG21–1*, *MiPG14*, *MiPG69–1*, *MiPG17*, *MiPG49*, *MiPG23–3*, *MiPG22–7*, and *MiPG16* were highly expressed during post-harvest fruit ripening, which may promote softening [38,101]. A total of 212 *MibHLHs* genes [102] and 315 *MiWD40s* genes were identified in the mango genome. Among them, MiTTG1 interacted physically with MiMYB0, MiTT8, and MibHLH1 in tobacco leaves [87], suggesting that a new ternary complex may be formed in mango, which may play an important role in plant defense regulatory network, development and other physiological processes.

With the development of metabonomics, secondary metabolites can undergo quantitative and qualitative analysis, emphasizing genes involved in metabolite synthesis. Current evidence suggests that *Mi9LOX* and *MiEH2* participate in lipid biosynthesis. The concentrations of δ-valerolactone and γ-decalactone significantly increased when *Mi9LOX* was overexpressed, and the concentrations of δ-valerolactone, γ-hexalactone, and δ-hexalactone increased when *MiEH2* was overexpressed, which further indicated that these genes might be involved in the biosynthesis of biogenesis of lactones from Alphonso mango [41]. It has been shown that the key structural genes of anthocyanin and proanthocyanidin synthesis in fruits, *MiCHS*, *MiANS*, and *MiUFGT1*, play an important role in the anthocyanin biosynthesis of mango peel. The MYB transcription factor regulates the expression of these genes. Compared with red mango (Guifei), green mango (Guiqi) and yellow mango (Jinghuang) produce fewer anthocyanins during maturity, secondary to the decrease in MiPAL activity at the translational level. It has been reported that the related transcription factors *MiWRKY1*, *3*, *5*, *81*, and *84* are upregulated during light-induced anthocyanin accumulation, indicating that these genes may regulate the biosynthesis of anthocyanins in mango [77–79]. *MiPSY*, *MiZDS*, *MiBCH*, and *MiZEP* regulated the synthesis of carotenoids, and the transcripts were positively correlated with the total carotenoid content, but there was no significant difference in the expression of CRTISO among varieties. In addition, the differentially expressed carotenoid catabolism genes may explain the heterogeneity in carotenoid content among the three mango varieties. The expression of carotenoid catabolic genes (*MiCCD1*, *MiNCED2*, and *MiNCED3*) decreased faster in ‘Kaituki’, resulting in higher carotenoid content in ‘Kaituki’ than in the other two varieties [80,81]. However, no studies have hitherto reported on MYB transcription factors that specifically regulate the synthesis of polyphenol metabolites.

Few studies have been conducted on other tissue parts of the mango fruit. *MiAUX1–4*, an early auxin response gene, and *MiPIN1,* an auxin polar transport carrier element, promote root formation in Arabidopsis transgenic plants [103]. In 2017, Denisov *et al.* verified the response of *MiLAX2* and *MiPIN1* to growth. In *A. thaliana*, MiERS1a and MiERS1a responded positively to the ethylene signal [84]. However, the key enzyme genes *MiACO* and *MiACS* in the ethylene biosynthesis pathway and ethylene signal-related transcription factor *MiERS1* could respond to salicylic acid and nitric oxide signals [85]. Without mango genome annotation, 18 complete *MieIFs* gene sequences were obtained through transcriptome data, and their expressions under salt stress, low-temperature stress and low-temperature stress were analysed. It was found that *MieIF1A-a*, *MieIF5*, and *MieIF3sB* might be candidate genes for improving the salt tolerance of mango [86]. Sixteen members of the *Mi14–3-3* gene family were identified from the ‘SiJiMi’ mango genome database. By analysing their expression patterns under drought, salt stress and low-temperature stress, it was found that the *Mi14–3-3* gene family played an important role under such stress conditions in mango [89].

At present, validation of gene function and the genetic transformation system of mango fruit have not been conducted due to low transformation efficiency and genotype dependence. Only some flowering regulatory and carotenoid synthesis genes have been validated in plant models, including *A. thaliana* and tobacco. Accordingly, it is necessary to find an effective transformation system for mango.

## Concluding remarks and future prospects

It benefited from the progress in sequencing technology, deciphering the mango genome and analysing fruit-related transcriptomes provide valuable data for variety identification, genetic diversity and transcriptional regulation of biological processes. In particular, flowering regulation, refining mango fruit appearance and growth characteristics has gained momentum. Future genome-wide association using multi-omics data, together with genetic population, natural population analysis and even accessions constructed pan-genomes will pinpoint regulatory genes for key traits, such as disease resistance genes, allowing easy and accurate breeding of new high-quality varieties in a short period of time, based on the focus on developing efficient mango tissue culture regeneration and gene editing technologies. These efforts may eventually bring a paradigm shift for mango breeding, which has substantial economic importance in tropical and subtropical regions.

## Author contribution

M.S. drafted the original manuscript. Critical inputs and corrections were successively provided by H.W., Z.F., and H.H. during the preparation process. H.M. is the project leader and helped in the conception and structure design of the manuscript and final proofing of the manuscript for submission.

## Data availability

Authors confirm the availability of data and that any required links or identifiers for data are present in the manuscript as described.

## Conflict of interest

The authors declare that the research was conducted in the absence of any commercial or financial relationships that could be construed as a potential conflict of interest.
